# Video game addiction is associated with early stage of inhibitory control problems: An event‐related potential study using cued Go/NoGo task

**DOI:** 10.1111/adb.13391

**Published:** 2024-04-02

**Authors:** Mazyar Fathi, Ali Mohammad Pourrahimi, Ahmad Poormohammad, Sara Sardari, Mohammad Amin Rajizadeh, Shahrzad Mazhari, Donya Pourkand

**Affiliations:** ^1^ Ibn‐e‐Sina and Dr Hejazi Psychiatry Hospital Mashhad University of Medical Sciences Mashhad Iran; ^2^ Kerman Neuroscience Research Center Kerman University of Medical Sciences Kerman Iran; ^3^ Kermanshah University of Medical Sciences Kermanshah Iran

**Keywords:** cued Go/NoGo, event‐related potential (ERP), inhibitory process, video game addiction

## Abstract

Video game addiction (VGA) is associated with cognitive problems, particularly deficits in inhibitory control. The present study aimed to investigate behavioural responses and event‐related potential associated with specific response inhibition using the cued Go/NoGo task to examine the effects of VGA on brain activity related to response inhibition. Twenty‐five individuals addicted to video games (action video games) and 25 matched healthy controls participated in the study. The results showed that the VGA group had significantly more commission error in the NoGo trials and faster reaction time in the Go trials compared with the control group. The event‐related potential analyses revealed significant reductions in amplitudes of N2 cue and N2 NoGo in the VGA group. While there was no significant difference between the N2 amplitudes of the Go and NoGo trials in the VGA group, the control group had a larger N2 amplitude in the NoGo trials. These results indicate that VGA subjects have difficulties in the early stages of response inhibition, as well as some level of impairment in proactive cognitive control.

## Graphical abstract

The graphical abstract depicts an overview of the study and main results. The main results demonstrated the problem in preparatory process, conflict monitoring, and response inhibition.

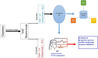

## 1 INTRODUCTION

Billions of people enjoy playing video games all over the world, but excessive video gaming could have adverse consequences for players and their families. The World Health Organization has added gaming disorder to its list of mental health conditions.^1^, ^2^ Gaming disorder, also known as video game addiction (VGA), is defined as the problematic and compulsive playing of video games that results in significant dysfunction in various life domains. This persistent or recurrent pattern of gaming behaviour is characterized by impaired control over gaming, excessive use despite negative consequences, withdrawal, and tolerance symptoms.^3^, ^4^ Excessive video game playing can have adverse effects on users' mental and physical health.^5^

Previous studies on behavioural addiction have paid close attention to dysfunction in inhibitory control. Response inhibition is the ability to refrain from engaging in behaviours that are currently inappropriate or not required, and it is essential for self‐regulation, adaptation, and controlling behaviour in different situations.^6^, ^7^, ^8^ Deficits in response inhibition are one of the predicting factors for different addiction types.^9^, ^10^, ^11^ There is some evidence that game addiction may share similar neural correlates as other neurocognitive deficits, such as obsessive‐compulsive disorder and attention deficit hyperactivity disorder.^12^, ^13^ For example, one study found that people with game addiction obsessive‐compulsive disorder showed analogous patterns of increased activity in the prefrontal cortex and striatum, which are areas involved in decision‐making, impulse control, and reward‐related processes.^13^ This finding suggests that game addiction may be driven by a reward system that is similar to the reward system that is involved in other addictions, such as drug addiction.^14^ Because individuals with VGA show some characteristics similar to substance dependence, it was hypothesized that there might be impaired inhibitory control in VGA subjects.^5^

The cued Go/NoGo task is a frequently used paradigm for evaluation of response inhibition, execution, and preparation.^15^ There are many differences between the cued Go/NoGo and the conventional Go/NoGo tasks. The main difference is that in cue Go/NoGo, participants are presented with a cue that indicates whether they should respond or not respond to a subsequent stimulus, while in conventional Go/NoGo, participants are only presented with the stimulus and must decide whether to respond or not respond without any cue. Moreover, in contrast to the conventional Go/NoGo task, the cue task contains equal probability of Go and NoGo trials that are presented after preparatory cue stimuli that create an uncertain context in which the inhibition is nonselective.^16^ Moreover, a defined cue indicates whether the subsequent stimulus may need a response or not.^15^, ^17^, ^18^ This evokes top‐down response preparation processes, facilitating speeded reactions.^19^

Event‐related potentials (ERPs) are a non‐invasive method with a high temporal resolution that has been suggested as a sensitive method to study response inhibition. By measuring the neural activity in the milliseconds, ERPs allow precise quantification of temporal characteristics of neural activity.^20^ The N2 component of ERP appears to index a cognitive control process in paradigms such as the visual Go/NoGo task. Previous studies have demonstrated that the N2 obtained from the cue is related to cognitive control, conflict detection, and preparation for inhibition or execution.^21^, ^22^, ^23^ P3 cue correlates to the attentional preparatory processes of cue orientation and reflects the attentional resources needed for the correct response to the expected target^24^, ^25^, ^26^ and assessment of stimulus.^19^, ^27^ In addition, the presentation of a second stimulus generates N2 Go potential, which is related to the allocation of attention to competing stimuli.^28^ P3 Go is related to the source of attention and updating task‐relevant information.^26^, ^29^ Centrally distributed P3 Go is related to assessment and classification of stimulus,^30^ and parietal P3 is an index of response selection.^31^ ERP components that are associated with conflict monitoring and response inhibition are N2 NoGo and P3 NoGo, which both have fronto‐central distribution.^26^ Moreover, it is suggested that N2 NoGo may have certain roles in perception and the cognition competition between Go and NoGo trials.^21^ Other studies suggest that N2 NoGo reflects some aspects of response inhibition and conflict monitoring,^32^ so it may be a specific indicator of response inhibition and cognitive control.^32^, ^33^ Some studies have proposed that P3 NoGo is related to the response inhibition process,^26^ while recent studies have suggested that P3 NoGo is more related to the evaluation of the inhibitory process and outcome of inhibition.^17^, ^34^, ^35^

Several studies have investigated inhibitory control in individuals with substance dependence and behavioural addiction by using Go/NoGo tasks. Cigarette smokers have shown smaller N2 NoGo and P3 NoGo amplitudes.^36^, ^37^, ^38^, ^39^ Yang et al. investigated response inhibition in heroin‐addicted individuals and observed a decreased N2 NoGo amplitude, while the P3 amplitudes were similar to controls.^40^ Littel et al. found no differences in N2 NoGo and P3 NoGo amplitudes between excessive internet users and controls, but other studies showed reduced N2 NoGo and increased P3 NoGo amplitudes.^41^, ^42^

However, many studies have evaluated response inhibition in the context of internet and gaming disorders using the conventional Go/NoGo task. These studies reported different results regarding the mechanisms of conflict monitoring, inhibitory control, N2 and P3 components, and the effect of impulsivity. For example, in gaming disorder subjects, one study showed that problems in inhibitory control were related to lower N2 NoGo amplitudes compared with the control group. However, another study suggested that the latency of N2 NoGo is related to problems in inhibitory control, and other studies have suggested a reduction in P3 NoGo amplitudes.^42^, ^43^, ^44^ These discrepancies in results may be related to differences in task difficulty across studies. These differences may cause changes in behavioural and electrophysiological inhibitory control parameters,^42^, ^43^, ^44^ as well as different effects on the cognitive system.^45^, ^46^, ^47^ Therefore, the results of these studies may provide an ambiguous picture of conflict and inhibition under VGA. To address these gaps and provide a clearer understanding of conflict and inhibition in the context of VGA, our study employed a visual version of the cued Go/NoGo task. This task allowed us to specifically investigate inhibition, preparatory processes, and execution in individuals with VGA who exclusively play action video games. This study aimed to investigate inhibition, preparatory processes, and execution in individuals with VGA who play action video games by using a visual version of the cued Go/NoGo task. N2 and P3 amplitudes of the cue were used to evaluate the preparatory process, and N2 and P3 amplitudes of Go and NoGo trials were used to evaluate execution and inhibition.

We hypothesized that individuals with VGA will exhibit impaired inhibitory control processes, specifically in the preparatory process and response inhibition, both behaviourally and electrophysiologically. This impairment may be reflected by a reduction in N2 amplitude, indicating deficits in the early stage of response inhibition. Furthermore, we considered the potential confounding effects of anxiety, depression, and impulsivity, which commonly co‐occur with VGA. By measuring these psychological parameters, we aimed to minimize any interference they may have with the observed ERP components.^19^, ^48^, ^49^

## 2 METHOD

### 2.1 Participants

A group of 25 individuals with VGA was recruited from two local game clubs. Inclusion criteria were as follows: male gender, aged between 17 and 35 years, playing action video games, playing 30 h or more online video games per week for at least 12 months, and having a score of 2.5 or higher on the VGA test (VAT).^50^, ^51^ The control group was composed of 25 male participants who were between 17 and 35 years of age. They were screened for game addiction and had a VAT score of less than 1.5.^51^ Exclusion criteria for all the participants included substance abuse (except for cigarette smoking), traumatic brain injury, psychological and neurological disorders, use of psychotropic medications, and a history of severe memory problems. To reduce the nicotine's effect on performance, smoker participants were asked not to smoke 1 h before the experiment.

All the participants were right‐handed and completed the following self‐reported questionnaire: VAT was used to assess game addiction intensity. It is a 14‐item self‐reported questionnaire, with a 5‐point Likert scale ranging from 0 to 4 (*never* to *very often*).^50^ Possible scores ranged from a minimum of 0 to a maximum of 4 (the sum of scores divided by 14), with higher scores indicating the severity of the disorder. The reliability (Cronbach alpha = 0.81) and validity (0.726) of these questions are acceptable. An example of a VAT item is “How often do you find it difficult to stop gaming.”^51^, ^52^ Barratt Impulsiveness Scale 11 contains 30 items that measure cognitive and motor impulsivity, for example, “1 plan tasks carefully,” with a 4‐point Likert scale ranging from 1 to 4. The range of scoring of this test is between 30 and 120 (Cronbach alpha = 0.83).^53^, ^54^

Beck Depression Inventory (BDI) was also used to assess depression. This questionnaire contains 21 items, with a 4‐point Likert scale ranging from 0 to 3, leading to final scores between 0 and 63, and acceptable reliability (Cronbach alpha = 0.81) and validity (0.80).^55^, ^56^ The Beck Anxiety Inventory (BAI) was used to assess anxiety. This questionnaire contains 21 items with a 4‐point Likert scale ranging from 0 to 3. The final scoring ranges from 0 to 63, and it has appropriate reliability (Cronbach's alpha = 0.72) and validity (0.83).^57^, ^58^

Handedness was determined based on the Edinburgh inventory, which contains 10 items scored from 1 to 5. The sum of the scores for the 10 items is considered the laterality score, which ranges from 10 (*always left*) to 50 (*always right*). The validity (98.5%) and reliability (Cronbach's alpha = 0.943) of this questionnaire have already been obtained.^59^, ^60^, ^61^

The study was approved by the Ethics Committee of Kerman University of Medical Sciences (Ethics code: IR.KMU.REC.1397.279).

### 2.2 Procedure assessment

The participants were tested in a soundproof and dimly lit room. They filled in the four questionnaires (VAT, BIS, BDI, and BAI) before taking part in the cued Go/NoGo tasks. To decrease ambient noise, all the experiments were recorded in a soundproof and dimly lit room, which satisfied the ANSI S3.1‐1999 standard. The participants sat on a comfortable chair with their heads fixed on a chin rest in a relaxed posture. The distance between the participants and a 17 in. monitor screen was 1.5 m. The task was designed by Psytask software, version 1.53.17 (Mitsar Inc., Russia).

### 2.3 Experimental task

**FIGURE 1:**
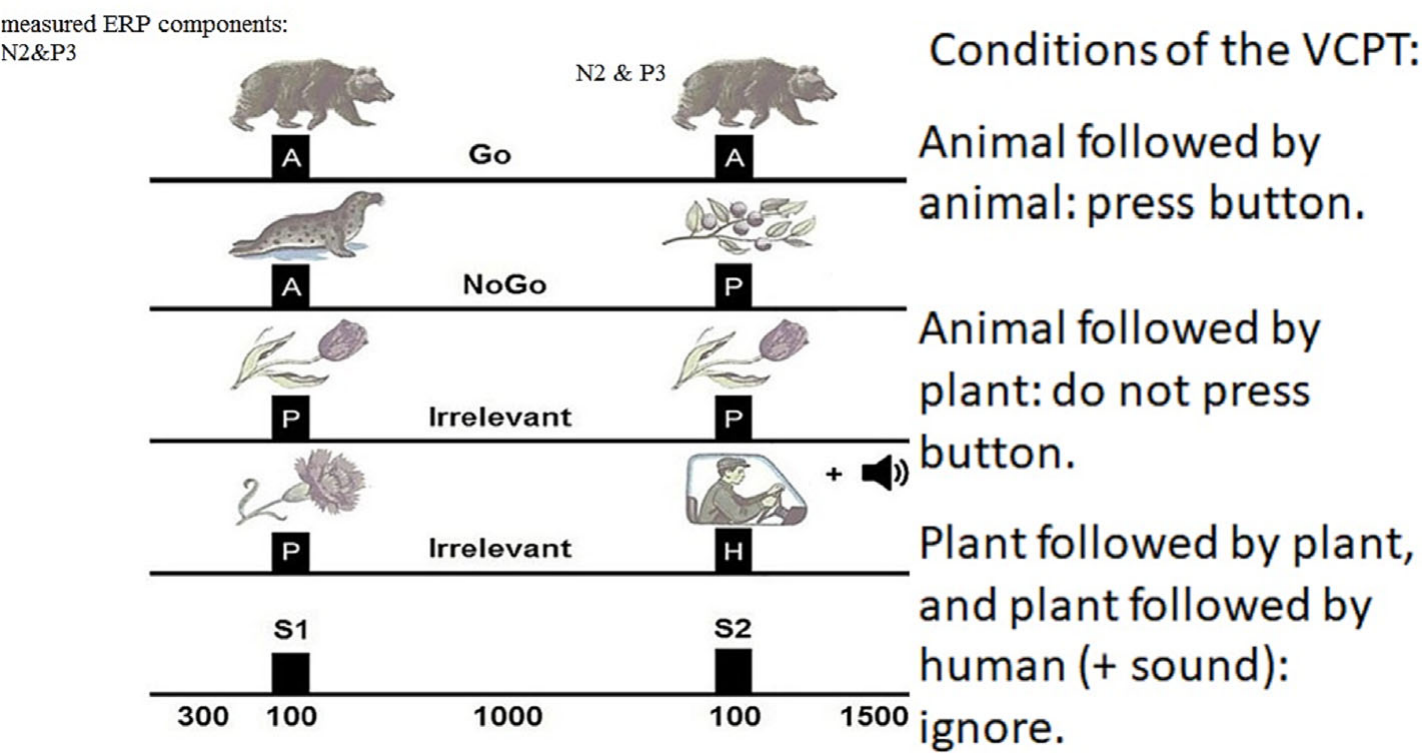
The cued Go/NoGo task. The total duration of the task was 3000 ms, S1 was presented after 300 ms, and S2 was presented 1400 ms after the onset of the trial. The duration of presentation for each stimulus was 100 ms. Go trials required the participants to respond to S2, but in NoGo trials, they needed to suppress a prepared action. Irrelevant trials should have been ignored and required no preparation for action. ERP, event‐related potential.

The cued Go/NoGo consisted of 400 pairs of images in three categories: animals, humans, and plants, with 20 different images in each category. Each trial consisted of a pair of stimuli (S1 and S2) in four combinations: animal–animal (A–A) as Go trial, animal–plant (A–P) as NoGo trial, and plant–plant (P–P) and plant–human as irrelevant trials (Figure 1). The images that were presented in (A–A) and (P–P) were identical. S1 and S2 stimuli were presented for 100 ms with a 1000 ms interstimulus interval, while the intertrial interval was 1500 ms.

The trials were grouped into four blocks separated by a short break. A unique set of five pictures from each picture category was selected in every block. Each block was composed of a pseudo‐random presentation of 100 stimulus pairs with equal probability for each trial category. The trials were divided into four blocks, each containing 100 trials with a unique set of images that were not similar to other blocks (there were five new images in each block) and the paired images were pseudo‐randomized so there were equal numbers of Go, NoGo, and irrelevant trials in each block. To maintain an adequate level of alertness and arousal in this task, the human stimuli (H) were paired with different 70 db sounds that consisted of five different frequencies (500, 1000, 1500, 2000, and 2500 Hz).^19^, ^62^ The sound stimuli were presented from two speakers that were placed at the ear level (distance from the head centre: 90 cm and hearing angle: ±90° azimuth). The Go stimulus was (A–A) and the NoGo stimulus was (A–P).

The participants were instructed to press a keyboard button (space bar) with their right hand's index finger to the (A–A) trial (Go), withhold their response to the (A–P) trial (NoGo), and ignore irrelevant trials (P–P and plant–human). The duration of the task was 20 min, and the participants had 2 min of rest after the first 10 min.

Omission error in the Go trial was defined as not responding to S2. Commission error in the NoGo trial was defined as a response to S2. The response was considered correct if it occurred between 100 and 1000 ms after stimulus presentation in the Go trial.

### 2.4 ERP acquisition

The Psytask software was used to present the task in this study, and it was synchronized with the WinEEG software to provide synchronous stimuli presentation and EEG recording. EEG was recorded and analysed by a 32‐channel Win EEG system (version 2.126.97, Mitsar Inc., Russia). The sampling rate was 500 Hz, and electrodes were positioned according to the international 10–20 system. Impedance was kept below 5 kΩ. Low‐pass and high‐pass filters were 0.1–45 Hz. EEG data were computed by Win EEG software and recorded using monopolar montage, and input signals were referenced to the linked ear.

ERPs were computed individually for the Go, NoGo, and irrelevant trials. Artefact correction was performed as follows: First, the raw EEG was visually inspected, and noisy trials (more than 100 μV) were removed manually. Second, eye blink artefacts were corrected by zeroing the activation curves corresponding to eye blinks. Third, the area with the minimum noise was selected as the sample, and independent component analysis was run, and then, independent component analysis components related to artefacts were removed from EEG data.^63^

The ERPs data were analysed after artefact rejection, “N2 cue” was measured as the second negative peak between 180 and 300 ms after presentation of cue (S1) at frontal electrodes, and “P3 cue” was defined as the largest peak between 300 and 550 ms after presentation of (S1) at central electrodes. N2 in the Go and NoGo trials were measured as the second negative peaks between 200 and 350 ms after the (S2) stimulus presentation at frontal electrodes, and P3s in the Go and NoGo trials were measured as the largest peaks in latency between 280 and 500 ms after the presentation of the second stimulus (S2) at central electrodes and were analysed for the three frontal and central electrodes (Fz, F3, F4, Cz, C3, and C4).^64^

## 3 STATISTICAL ANALYSIS

Independent samples *t*‐test were used for comparing demographics, psychometrics, and cued Go/NoGo task performance parameters (reaction time and commission and omission errors) between VGA and the control group. A mixed effects two‐way repeated measures analysis of variance was conducted for each ERP with two within‐subject factors: (i) trial type (Go, NoGo, and irrelevant trials) and (ii) electrode locations (Fz, F3, F4, Cz, C3, and C4). Significant main effects, interactions, and follow‐up pairwise comparisons were examined after adjustment for multiple testing (Bonferroni). Pearson's correlation coefficients were applied to calculate the correlation between VAT, HOURS/DAY, and REPs parameters. All the analyses were conducted using the Statistical Package for the Social Sciences software, version 20.

## 4 RESULTS

Table 1 shows the demographical data in which the two groups were not significantly different in terms of age, years of education, and scores of Edinburgh, BDI, BAI, and BIS (all *p* > 0.05). As expected, the VGA group had significantly higher VAT scores (*p* ≤ 0.0001) and spent significantly more time (*p* = 0.002) and days (*p* ≤ 0.0001) playing video games. Relevant data were reported with details in our previously published articles.^65^

**TABLE 1:** Demographic and clinical characteristics of the study participants (mean ± standard deviation).

Variable	VGA	Controls	*p*‐value
Age (year)	20.39 ± 3.03	19.91 ± 1.94	0.45
Edinburgh	78.19 ± 14.84	75.74 ± 22.28	0.59
BDI	16.00 ± 11.64	16.44 ± 10.95	0.87
BAI	15.42 ± 12.12	15.45 ± 12.05	0.99
BIS‐11	65.26 ± 11.30	64.59 ± 7.51	0.78
VAT	2.82 ± 0.28	1.05 ± 0.62	<0.00
Day/week	6.50 ± 0.77	2.75 ± 2.12	<0.000
Hours/day	5.72 ± 1.24	1.34 ± 1.11	<0.000

Abbreviations: BAI, Beck Anxiety Inventory; BDI, Beck Depression Inventory; BIS‐11, Barratt Impulsiveness Scale 11; Day/week, number days per week spent gaming; Hours/day, number of hours per day spent gaming; VAT, video game addiction test; VGA, video game addiction.

### 4.1 Behavioural data

Table 2 shows the behavioural data on the cued Go/NoGo task of the two groups. In the Go trials, the omission error was not significantly different between the two groups. In the NoGo trials, the commission error differed significantly between the two groups, and the VGA group had more commission errors than the control group (*p* = 0.027).

**TABLE 2:** Behavioural results on the cued Go/NoGo task for video game addiction (VGA) and control groups.

Variable	VGA	Control	*p*‐value
OM‐Go (mean error)	2.08 ± 3.558	1.48 ± 1.388	0.436
COM‐NoGo (mean error)	0.96 ± 0.935	0.44 ± 0.651	0.027
RT (ms)	352.36 ± 65.68	415.08 ± 88.55	0.007

Abbreviations: COM‐NoGo, number of commission errors in NoGo trials; OM‐Go, number of omission errors in Go trials; RT, reaction time.

Regarding reaction time, the results showed a significant difference between the two groups, that is, the VGA group performed significantly faster than the control group)*p* = 0.007(.

### 4.2 Electrophysiological data

**FIGURE 2:**
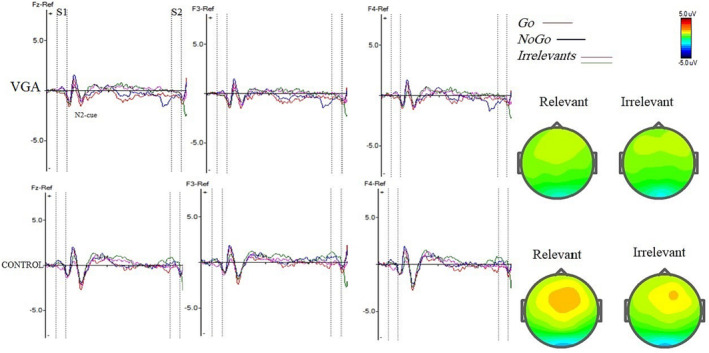
Grand average event‐related potential waves over frontal electrodes for the video game addiction (VGA) and the control subjects in relevant cue (animal = S1) and irrelevant cue (plant = S1) conditions in the cued Go/NoGo task and N2 components at Fz, F3, and F4 electrodes in VGA and control groups. The vertical dotted lines show the duration of S1 and S2. The topographic voltage maps of the grand averages are displayed for conditions (228–240 ms) at Fz, depicting N2‐relevant and N2‐irrelevant. The colour scale represents microvolts, with red and blue indicating the highest and lowest voltages, respectively. The colour progression follows the order: red > yellow > green > blue, illustrating voltage variations across the scalp.

**FIGURE 3:**
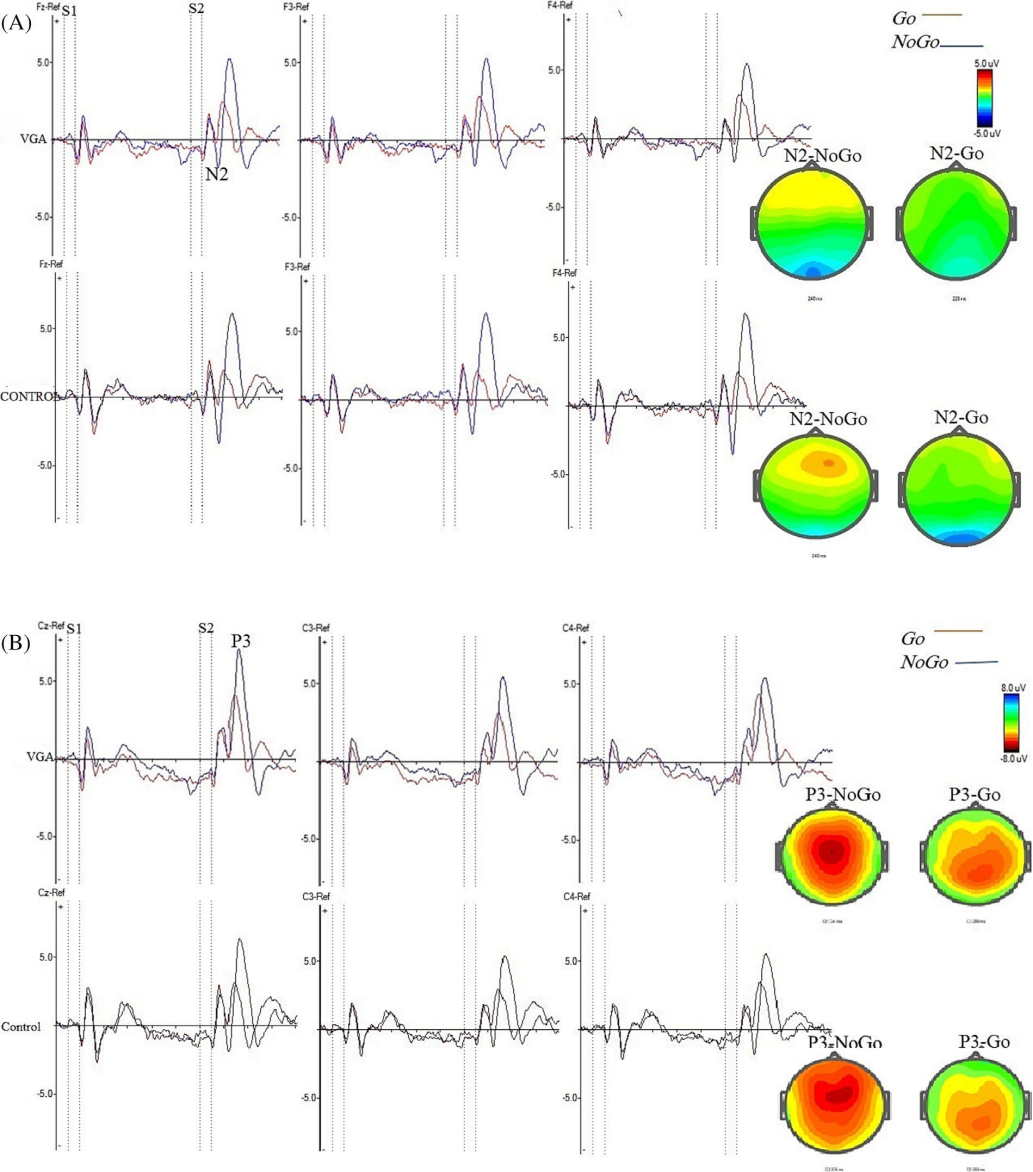
Grand average event‐related potential waves over frontal and central electrodes for the video game addiction (VGA) and the control groups after S2 in Go (A–A) and NoGo (A–P) conditions in the cued Go/NoGo task. (A) N2 components at Fz, F3, and F4 electrodes in VGA and control groups. (B) P3 components at Cz, C3, and C4 electrodes in VGA and control groups. The topographical voltage maps of the grand averages are presented for N2‐NoGo and N2‐Go conditions (230–248 ms) at Fz, as well as for P3‐Go and P3‐NoGo conditions (290–328 ms) at Cz. The colour scale represents microvolts, with red and blue indicating the highest and lowest voltages, respectively. The colour progression follows the order: red > yellow > green > blue, illustrating voltage variations across the scalp.

Table 3 shows the mean amplitudes and latencies of N2 and P3 for each stimulus of the VGA and control groups. The grand averages of N2 and P3 for each group are demonstrated in Figures 2 and 3.

**TABLE 3:** Mean event‐related potential amplitudes (μV) for video game addiction (VGA) patients and control group.

	VGA	Control		VGA	Control	
	Relevant trials			Irrelevant trials		
	Mean ± *SD*	Mean ± SD	*p*‐value	Mean ± *SD*	Mean ± *SD*	*p*‐value
N2 cue						
Fz	−1.74 ± 1.7	−3.66 ± 1.80	0.001	−1.88 ± 1.9	−2.81 ± 1.37	0.075
F3	−1.55 ± 1.68	−2.75 ± 1.12	0.009	−1.61 ± 1.22	−2.19 ± 1.04	0.101
F4	−1.16 ± 1.60	−2.64 ± 1.50	0.004	−1.50 ± 1.44	−2.27 ± 1.35	0.081
P3 cue						
Cz	2.32 ± 1.49	2.70 ± 1.30	0.201	1.42 ± 1.12	1.90 ± 1.45	0.399
C3	1.87 ± 1.26	2.21 ± 0.92	0.133	1.14 ± 0.67	1.56 ± 1.05	0.337
C4	2.27 ± 1.39	2.31 ± 1.37	0.579	1.57 ± 1.07	1.76 ± 0.98	0.986
N2 Go						
Fz	−0.30 ± 2.80	−1.59 ± 2.05	0.074			
F3	−0.98 ± 2.28	−1.07 ± 1.47	0.874			
F4	−0.23 ± 2.45	−1.03 ± 1.61	0.189			
N2 NoGo						
Fz	−1.42 ± 2.70	−2.91 ± 2.15	0.040			
F3	−1.01 ± 2.35	−2.03 ± 1.97	0.111			
F4	−0.89 ± 2.25	−2.53 ± 1.71	0.007			
P3 Go						
Cz	5.23 ± 3.15	4.43 ± 2.48	0.333			
C3	4.14 ± 2.43	4.14 ± 2.54	1.000			
C4	5.09 ± 3.33	4.34 ± 2.38	0.372			
P3 NoGo						
Cz	8.35 ± 4.44	7.60 ± 4.24	0.553			
C3	6.65 ± 3.33	6.52 ± 3.46	0.902			
C4	6.89 ± 3.65	6.71 ± 3.53	0.864			

*Note*: Group means and standard deviations (*SD*s) are reported.

### 4.3 ERPs of cue (S1)

Repeated measure analysis of variance showed that there was no significant main effect of the trial on the amplitude of N2 (F (1, 40) = 1.742, *p* = 0.194, ŋ2 = 0.042), while there was a significant main effect of group on N2 amplitude (F (1, 40) = 8.750, *p* = 0.005, ŋ2 = 0.179). Follow‐up analyses showed that the VGA group had a significantly smaller N2 amplitude relative to the controls in the relevant cue, which was the smallest at the Fz electrode (*p* ≤ 0.01). Moreover, there was a significant interaction between group and trial (F (1, 40) = 5.934, *p* = 0.019, ŋ2 = 0.129), in that the N2 component was more negative in the relevant trials compared with the irrelevant trials, only in the control group. There was no significant difference between the two groups in terms of N2 latency (F (1, 42) = 0.285, *p* = 0.596, ŋ2 = 0.007), and trial's effect on latency of N2 was not significant (F (1, 42) = 3.717, *p* = 0.061, ŋ2 = 0.081).

The analyses showed that there was a significant main effect of trial on the amplitude of P3 cue, reflecting that the amplitudes in the relevant cues were larger compared with the irrelevant cues across the groups (F (1, 47) = 242.599, *p* ≤ 0.001, ŋ2 = 0.979). However, there were no significant main effects of group on P3 cue amplitude (F (1, 47) = 1.48, *p* = 0.229, ŋ2 = 0.035). Similarly, no significant effects of trial (F (1, 45) = 3.80, *p* = 0.965, ŋ2 = 0.78) or group (F (1, 45) = 0.373, *p* = 0.544, ŋ2 = 0.008) were observed on P3 latency.

### 4.4 ERPs of stimulus 2 (S2)

The analyses showed that there was no significant difference between the VGA and control groups in terms of N2 amplitude of the Go trials (F (1, 46) = 1.552, *p* = 0.219, ŋ2 = 0.033). In contrast, group had a significant main effect on N2 amplitude in the NoGo trials (F (1, 46) = 6.133, *p* = 0.017, ŋ2 = 0.118), in that the N2 amplitude of the NoGo trial was smaller in the VGA group compared with the control group, with the largest difference being in F4 electrode. In addition, the trial's effect was significant on N2 amplitude (F (1, 46) = 8.086, *p* = 0.005, ŋ2 = 0.161) and analysis showed that N2 amplitude was larger (more negative) in the NoGo compared with the Go trials in the control group, and the most significant effect was found in F4 electrode (*p* = 0.003). There was no significant difference between the groups in terms of latency in the Go (F (1, 46) = 1.552, *p* = 0.219, ŋ2 = 0.033) and the NoGo trials (F (1, 46) = 0.424, *p* = 0.518, ŋ2 = 0.009).

The model indicated that the trial had a significant main effect on P3 amplitude (F (1, 46) = 30.224, *p* ≤ 0.001, ŋ2 = 0.397), and follow‐up analyses suggested that P3 amplitude was larger in the NoGo trials compared with the Go trials in both groups. But the analyses failed to reveal any significant main effect from group on P3 amplitude in the Go (F (1, 45) = 1.262, *p* = 0.267, ŋ2 = 0.027) and NoGo (F (1, 45) = 0.03, *p* = 0.945, ŋ2 = 0.001) trials.

No significant difference was observed between the VGA and control groups in terms of P3 latency in both Go (F (1, 45) = 0.48, *p* = 0.828, ŋ2 = 0.000) and NoGo (F (1, 45) = 0.104, *p* = 0.749, ŋ2 = 0.002) trials. Moreover, trial's effect was not significant on P3 latency (F (1, 45) = 0.48, *p* = 0.542, ŋ2 = 0.008).

### 4.5 Correlation analysis

The findings indicated that there were no significant correlations between the VGA score and the ERP parameters within the VGA group, as illustrated in Table 4.

**TABLE 4:** Correlation between video game addiction test (VAT), H/P, and event‐related potential parameters.

Correlations		*R*	*p*‐value
VAT	N2 cue	0.40	0.54
	N2 NoGo	0.30	0.174
H/P	N2 cue	0.075	0.74
	N2 NoGo	0.045	0.84

Abbreviations: H/P, number of hours per day spent gaming; *R*, correlation coefficient.

## 5 DISCUSSION

**FIGURE 4:**
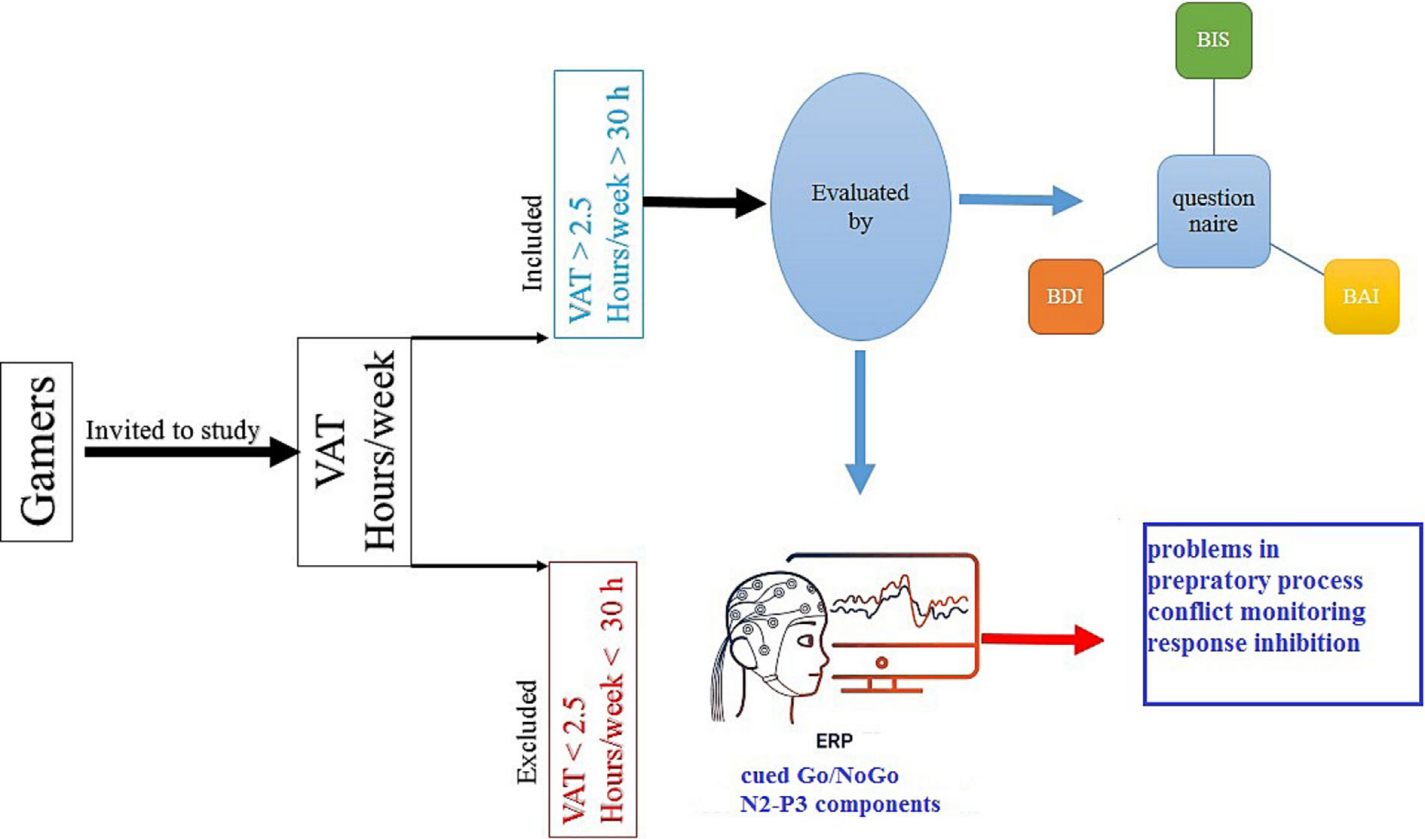
A schematic diagram with an overview of the study design and the main procedures. BAI, Beck Anxiety Inventory; BDI, Beck Depression Inventory; BIS, Barratt Impulsiveness Scale; ERP, event‐related potential; VAT, video game addiction test.

The purpose of this study was to examine the effects of VGA on behavioural and neurophysiological preparatory processes, response inhibition, and execution. The behavioural results showed that the VGA group made more commission errors in the NoGo trials and had faster RTs compared with the control group. The ERP analyses revealed significant reductions in amplitudes of N2 cue and N2 NoGo in the VGA group. Additionally, there was no significant difference between the N2 amplitudes of Go and NoGo trials in the VGA group, while a larger N2 amplitude was found in the NoGo trials in the control group (Figure 4).

### 5.1 Performance in the cued Go/NoGo task

Results showed that the VGA group made more commission errors in the NoGo trials. These findings are consistent with previous studies^50^ that had found more commission errors in internet game addicted individuals, excessive computer gamers, and pathological internet users.^44^ In another study, Luijten et al. showed that excessive gamers had more errors in the NoGo trials, which was associated with higher impulsivity.^50^ In general, higher commission errors in the VGA group may indicate difficulty in response inhibition.^66^

Consistent with previous studies, subjects with VGA had shorter reaction times than the controls.^67^, ^68^ However, what was contrary to other studies was that RT was not different between internet gaming disorder and control groups,^41^ indicating that this difference may be related to the type of games they play, which might have different effects on the cognitive system. For example, action video gamers need rapid motor movement, while puzzle gamers need slow and deliberate response.^46^, ^47^ It should be noted that researchers have shown that subjects with shorter RTs had weaker accuracy and more commission errors in cued Go/NoGo tasks. When a quick response is prioritized, it may decrease response control, and the response becomes more prone to errors.^62^

### 5.2 Electrophysiological performance in the cued Go/NoGo task

The ERP results demonstrated that in the VGA group, the amplitude of the N2 cue was attenuated in the relevant trials compared with the control group. Moreover, the amplitude of the N2 cue was more negative in relevant trials compared with irrelevant trials in the control group, but no significant difference was found in the VGA group. Some studies have suggested that a larger N2 amplitude is linked to better cognitive control in preparation for a prepotent response and better allocation of cognition to a prepotent response.^19^, ^21^ Therefore, a smaller N2 amplitude in the VGA group may indicate poorer conflict monitoring and response preparation. The similarity between N2 cues in relevant and irrelevant trials indicates that VGA subjects have a similar strategy in anticipating targets and conflict monitoring. It has been proven that accurate performance in tasks depends on accurate representation of context information and maintaining the data in the delay between the cue and the target.^69^ This response strategy, named *proactive cognitive control*, is triggered by contextual cues, which occur before goal‐relevant events and convey information to promote behavioural responses.^70^ Therefore, deficits in preparatory processes in VGA subjects may show the negative effects of VGA on proactive cognitive control. Bailey et al. showed that some ERP components (the medial frontal negativity and the frontal slow wave), which are related to proactive cognitive control, were attenuated in excessive gamers.^71^ Moreover, the functions anterior cingulate and lateral frontal cortex, which are related to proactive cognitive control, were changed in video gamers.^71^

The present findings indicated that the N2 amplitude in the NoGo trials was reduced in the VGA group compared with the control group. In addition, the N2 amplitude in the NoGo trials was larger than that of the Go trials in the control group, but not in the VGA group. Similarly, studies have shown that pathological internet users have smaller N2 NoGo amplitude than controls.^43^, ^72^ Nevertheless, another study showed no significant difference regarding N2 amplitude in the NoGo trials in subjects with internet game addiction.^41^ N2 in the Go/NoGo task has been accepted as the neurophysiological marker of inhibitory control.^73^, ^74^ In the Go/NoGo paradigm, inhibition of response to NoGo trials needs more cognition monitoring and intensifies the conflict, which is accompanied by creating a larger N2 NoGo.^75^, ^76^, ^77^ N2 is related to earlier stages of inhibition and conflict monitoring.^41^ The sources of N2 NoGo are the anterior cingulate and medial orbitofrontal cortex, which are related to conflict monitoring and inhibitory control.^73^, ^78^ Many structural studies on the experience of video gaming have suggested that there is a negative correlation between the functions of the anterior cingulate and orbitofrontal cortex.^3^, ^71^, ^79^, ^80^ Together, previous studies and the results of the current work suggest that the reduced inhibitory control in individuals with VGA could be related to a dysregulation in activation of inhibitory function at an early stage of cortical processing, while the later stages of inhibitory processing remain intact. This finding indicates that individuals with VGA have deficits in action inhibition and conflict monitoring. Overall, the smaller amplitude of N2 in the equal‐probability Go/NoGo task showed a deficit in neurophysiological mechanisms involved in an early stage of inhibitory control, which is confirmed with commission errors in the NoGo trials and may indicate a deficit in inhibitory control in the VGA subjects.

The difference in N2 NoGo between the VGA and control groups was observed in F4 electrode. In line with this study, Liu et al. showed the hypofunction of the right hemisphere of the brain in internet gaming disorder subjects using fMRI.^81^ Lianekhammy et al. demonstrated that there is a frontal asymmetry in violent video game players by an EEG study.^82^ The right hemisphere has a dominant role in response inhibition.^83^ Together, these results indicate that there might be a deficit in the right hemisphere's function that is associated with problems in inhibitory control in individuals with VGA.

On P3 component, the results of this study showed no significant difference in amplitude of P3 between VGA and control groups in the Go and NoGo trials. This result is in contrast with other studies that had shown differences in P3 amplitude in NoGo trials.^84^ In line with this study, Littel et al. found no significant difference in P3 amplitude in Go/NoGo paradigm between excessive computer game players and the control group.^42^ These results may show that VGA subjects do not have any problems in the late stage of inhibition.

The correlation analysis revealed no significant correlation between the VGA score and ERP waves. However, the correlation between VAT and N2 NoGo exhibited near‐marginal significance. These findings align with a previous study, which also reported no significant correlation between the internet addiction scale and ERP waves in gaming disorder subjects.^85^ Additionally, another study found no significant correlation between addiction score and ERPs (N2 and P3) in adolescents with internet gaming disorder.^49^ Similarly, our study demonstrated no significant correlation between time spent gaming and ERPs. These results are consistent with another study that did show a significant correlation between ERPs (error‐related negativity), but not N2 and P3 components.^42^

As a final note, the aim of this study was to investigate neurophysiological and behavioural performance using the cued Go/NoGo task. In conclusion, behavioural data indicated that VGA subjects had difficulties in response inhibition. ERP results demonstrated deficits in N2 cue, which may show that VGA subjects have problems in preparatory processes that can be considered as a deficit in proactive cognitive control. In addition, reduced N2 NoGo parameters in the VGA group are indicative of problems in the early stages of response inhibition and deficit in conflict monitoring, which are confirmed by more errors in the behavioural data.

### 5.3 Limitation and future study

This research has several limitations that can be considered in future studies. The finding of this research showed more commission errors and faster RT, which in future researches can be studied in terms of speed–accuracy trade‐off strategy. Moreover, in this study, only action video gamers were compared with the control group, but future researches are needed to compare different type of game users in term of inhibitory control. Furthermore, the correlation analysis revealed a near‐marginal significance difference between VAT and N2 NoGo. However, it is important to note that future research should aim to measure these correlations with a larger sample size.

## AUTHOR CONTRIBUTIONS

**Mazyar Fathi**: Study design; analysis; writing—original draft. **Ali Mohammad Pourrahimi**: Methodology; task design; editing. **Ahmad Poormohammad**: ERP analysis; methodology; rewriting draft. **Sara Sardari**: Software; investigation. **Mohammad Amin Rajizadeh:** Review and editing. **Shahrzad Mazhari**: Funding acquisition; supervision; writing. **Donya Pourkand**: Review; rewriting; language editing.

## CONFLICT OF INTEREST STATEMENT

The authors declare that they have no known competing financial interests or personal relationships that could have appeared to influence the work reported in this paper.

## ETHICS STATEMENT

The study was approved by the Ethics Committee of Kerman University of Medical Sciences (Ethics code: IR.KMU.REC.1397.279).

## Data Availability

The data that support the findings of this study are available on request from the corresponding author. The data are not publicly available due to restrictions (e.g., their containing information that could compromise the privacy of research participants).
